# Elicitation of the Acoustic Change Complex to Long-Duration Speech Stimuli in Four-Month-Old Infants

**DOI:** 10.1155/2015/562030

**Published:** 2015-12-22

**Authors:** Ke Heng Chen, Susan A. Small

**Affiliations:** University of British Columbia, Vancouver, BC, Canada V6T 1Z3

## Abstract

The acoustic change complex (ACC) is an auditory-evoked potential elicited to changes within an ongoing stimulus that indicates discrimination at the level of the auditory cortex. Only a few studies to date have attempted to record ACCs in young infants. The purpose of the present study was to investigate the elicitation of ACCs to long-duration speech stimuli in English-learning 4-month-old infants. ACCs were elicited to consonant contrasts made up of two concatenated speech tokens. The stimuli included native dental-dental /dada/ and dental-labial /daba/ contrasts and a nonnative Hindi dental-retroflex /daDa/ contrast. Each consonant-vowel speech token was 410 ms in duration. Slow cortical responses were recorded to the onset of the stimulus and to the acoustic change from /da/ to either /ba/ or /Da/ within the stimulus with significantly prolonged latencies compared with adults. ACCs were reliably elicited for all stimulus conditions with more robust morphology compared with our previous findings using stimuli that were shorter in duration. The P1 amplitudes elicited to the acoustic change in /daba/ and /daDa/ were significantly larger compared to /dada/ supporting that the brain discriminated between the speech tokens. These findings provide further evidence for the use of ACCs as an index of discrimination ability.

## 1. Introduction

One of the early steps for infants to learn a language is to recognize the phonetic distinctions and sound patterns of their native language. Infants with hearing loss are at risk for delays in speech and language development because they might not have access to important auditory cues during a period of rapid development in the first years of life. These early processes are complex and not fully understood in infants with normal hearing or limited auditory experience. One challenge in this area of research is the lack of a reliable, age-appropriate tool to assess an individual infant's capacity to detect and discriminate speech sounds. Such a tool would be invaluable in the clinic to assess hearing-aid benefit in infants in terms of ability to discriminate consonant and vowel contrasts. Because behavioural methods provide limited information about perceptual capacities and their underlying mechanisms, particularly in individual infants, cortical auditory-evoked potentials (CAEPs) offer a useful complement to behavioural measures. The slow cortical response, or P1-N1-P2, which is elicited to the onset of a stimulus has been studied extensively in adults and in some depth in infants to assess detection of speech sounds, although there are gaps in our understanding of these responses in infants, especially those with hearing loss who are aided. The acoustic change complex (ACC) which is elicited to a change in an ongoing stimulus has been a focus of recent research because it is thought to measure discrimination at the level of the auditory cortex which can provide insight into the brain's capacity to process the acoustic features of speech [1–3]. The ACC has also been well studied in adults but only a few studies to date have attempted to elicit the ACC in infants [4–7].

Research findings have suggested that the ACC may have the potential to be used as a clinical tool for assessing speech perception capacity. The ACC has been recorded in adults in response to speech stimuli such as consonant-vowel syllables, in which the acoustic change included frequency, amplitude, and periodicity cues similar to those found in normal conversational speech [1–3]. Ostroff et al. [3] investigated cortical potentials in response to the naturally produced speech syllable /sei/ and a typical N1-P2 complex has been recorded to the acoustic change from /s/ to /ei/. The finding suggests that the ACC reflects changes of cortical activation caused by amplitude or spectral change at the transition from consonant to vowel and it may have the potential to demonstrate discrimination capacity. Changes from aperiodic to periodic stimulation have also produced changes in cortical activation that contribute to the observed response. The ACC has also been used to measure intensity discrimination [8] and frequency discrimination [9, 10] with results commensurate with behavioural findings. Another important feature of the ACC is that it has been shown to have excellent test-retest reliability in adults to natural speech stimuli [11]. The ACC was also shown to be efficacious in individuals with sensorineural hearing loss [12] and in those with cochlear implants [12]. Furthermore, when compared to other ERPs, such as the MMN, the ACC elicits responses with larger amplitudes and better signal-to-noise ratios, thus requiring less time and fewer stimulus presentations for recording [2]. These advantages can be very important for testing infants and other populations that are difficult to test.

Small and Werker [4] first recorded ACCs in infants in response to English and Hindi consonant contrasts. More recently, Martinez and colleagues [5] also recorded ACCs to vowel contrasts in a small number of young children with normal hearing and hearing loss (aided and unaided). Small and Werker [4] presented 4-month-old English-learning infants with speech contrasts generated from a synthetic place-of-articulation continuum: a native dental-dental contrast /dada/, a dental-labial contrast /daba/, and a nonnative Hindi dental-retroflex contrast /daDa/. These stimuli were the same as those used in a behavioural study by Werker and Lalonde [13] that showed that infants successfully discriminated native /daba/ and nonnative Hindi /daDa/ contrasts when they were under 6 months of age but not when they were 10 months of age, when their responses were similar to adults. These findings support the hypothesis that infants are born with a biological predisposition allowing them to discriminate the universal set of phonetic contrasts, then a decline or reorganization in this universal phonetic sensitivity takes place by the end of the first year as a function of linguistic experience of the ambient language [14]. Consistent with the behavioural data, Small and Werker [4] reported that robust ACCs were elicited in most infant participants to /daba/ with P1, N1, and P2 components, but fewer infants had the different components of the ACC present in their responses to /dada/ and /daDa/ and the morphology of these ACCs was more variable compared to /daba/. The ACC was recorded infrequently to English /dada/ presumably because no distinct acoustic change between the two speech tokens was detected. This study did not replicate the behavioural findings for nonnative stimuli (i.e., elicitation of the ACC to nonnative stimuli /daDa/ in the 4-month-old infants). One possible cause for the inconsistency between the ACC and behavioural findings posed by the authors was that the relatively short stimulus length might not have been long enough to accommodate the neural refractory periods in the immature brain in response to all stimuli.

Infant responses are frequently biphasic waveforms with a large positive peak and following negativity [15–18] due to immaturities in the underlying neurogenerators. The infant responses reported by Small and Werker [4] resemble the P1-N1-P2 complex recorded in adult participants in terms of the morphology of the waveforms but there are obvious differences in the relative prominence of the peaks and significantly prolonged latencies relative to the adults due to immaturity. These findings are similar to other studies that reported more complex waveforms in infants when the duration and complexity of the stimuli are increased and the stimulation rate is decreased [19–25]. There is some debate over which cortical components dominate at which age [26–32]. Some studies indicate that the large positive peak is the most predominant peak in early childhood (1–4 years) [33], and the negative trough that follows it becomes increasingly robust (3–6 years) and dominates the cortical response until adolescence. Some researchers claim that the earlier negative component (N1 in adults) can be recorded in addition to the negative trough from about three years of age with slow stimulation rate [34, 35], while others suggest that N1 can only be reliably evoked when children reach 9–13 years [29, 31]. It is also important to note that the labelling of the large positivity and following negativity in infant waveforms by different research groups is somewhat arbitrary. For example, some groups use the adult labels, P2 and N2, to describe these features [4, 17, 22]; others use P1 and N2 or N450 [5, 18, 36]. For the purposes of the present study, N1-P1-N1-P2 were used for convenience to describe the infant waveforms recorded.

Interstimulus interval (ISI) is thought to have substantial effects on the morphology, amplitude, and scalp distribution of the cortical response, particularly in immature auditory systems. For example, increasing the ISI up to at least 10 s results in larger amplitudes of N1 and P2 and their magnetic counterparts [35, 37]. When the ISI is decreased to less than 300 ms, the amplitude of N1 is usually diminished and may not be readily detected in some cases [38]. Some researchers suggest that an adult-like N1 component can only be recorded in children with ISIs longer than 1 s [19, 34]. An earlier study reported that a systematic decrease in the latency of the N1 component occurred with an increase in ISIs from 250 to 1000 ms for children aged 9–13 years, but not for adults [34]. Because the refractory properties of underlying neural components involved in N1 response may not have been fully developed in children, this finding may have resulted from prolonged N1 recovery cycles overlapping with robust P1 and N2 peaks.

Only a few studies have studied the effects of stimulus and presentation parameters on infant cortical responses. Golding et al. [18] recorded cortical responses dominated by a large positive peak (P1) when they presented /m/ and /t/ to 7-month-old infants using ISIs that varied from 750 to 1500 ms. They found a modest decrease in P1 amplitude and no change in latency with decreased ISI; Sharma et al. [36] found the same pattern of results for 10- and 20-week-old infants for ISIs of 910 to 4550 ms. Golding et al. [18] also investigated the effects of stimulus duration on infant cortical responses and found that decreasing stimulation duration from 31 to 79 ms resulted in a small decrease in amplitude but no change in latency.

Although the stimulus duration (282 ms for each speech token) and the ISI (2200 ms) used by Small and Werker [4] were adequate for eliciting the ACCs to the native speech contrast /daba/ in infants, the neural population in the infant brain may need a longer time to recover from the initial firing in a more challenging test condition (i.e., the nonnative /daDa/ condition). Linguistic experience with their ambient language may also have already played a role in the development of speech perception, such that the 4-month-old infant brain found it more difficult to discriminate the nonnative /daDa/, even though they were expected to discriminate the contrast behaviourally. A longer stimulus duration could potentially compensate for the longer refractory period needed by young infants and be more optimal for elicitation of an ACC.

The purpose of the present study was to investigate the effect of long-duration speech stimuli on the ACC elicited in young infants to native and nonnative consonant contrasts. By allowing more time for neural refractoriness, it is expected that ACCs will be recorded in response to the changes in both native and nonnative speech contrasts with better morphology than previously reported by Small and Werker (2012).

## 2. Materials and Methods

### 2.1. Participants

Ten adult (mean age: 29 years; range: 24 to 38 years) and 24 infant (mean age: 4 months; 11 days; range: 4 months; 0 days to 5 months; 15 days) participants with normal hearing were included in this study. Adults were recruited from the community; infants were recruited through a database managed by the Infant Studies Centre at the University of British Columbia. All adults were English-speaking and all infants were learning English as a first language and had no exposure to the Hindi language. Infant participants were screened for hearing using transient-evoked otoacoustic emission (TEOAE) with the Madsen AccuScreen Pro (GN Otometrics). One infant who did not pass the hearing screening was excluded from the study. Nine infant participants were also excluded because data collection was not completed due to crying, excessive movement, or technical problems.

### 2.2. Stimuli

**Figure 1:**
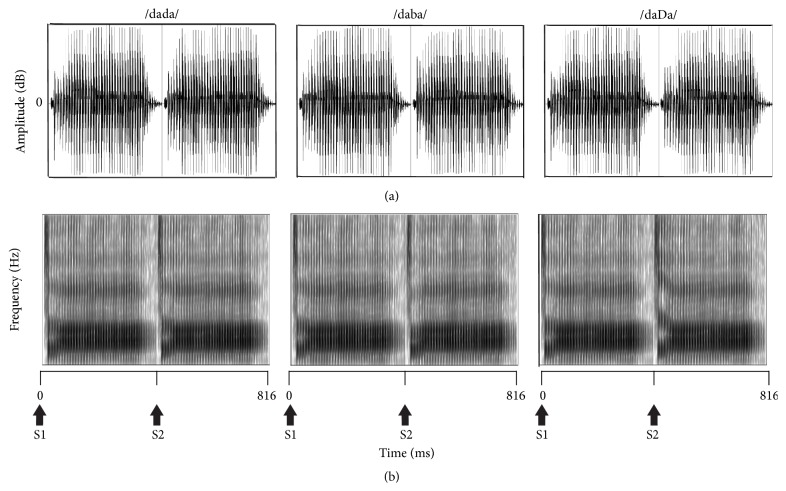
Stimuli used to elicit the acoustic change complex shown as waveforms in the time domain (a) and spectrograms (b). S1 and S2 indicate the time point where the first speech token /da/ and second speech tokens /da/, /ba/, and /Da/ begin.

The stimuli used in the present study were similar to those used by Small and Werker [4] and are shown in Figure 1. They were created from three speech tokens: English bilabial /ba/, dental /da/, and Hindi retroflex /Da/, which were selected from a synthetic voiced place-of-articulation continuum that was originally constructed to examine the perception of retroflex and dental-stop consonants in infants [13]. The three speech tokens were paired to form speech contrasts containing acoustic changes from /da/ to /ba/ and from /da/ to /Da/ (i.e., /daba/ and /daDa/) with no gap in between. Both speech contrasts started with the same token /da/ (denoted as S1), which was followed by one of the other two tokens /ba/ or /Da/ (denoted as S2). A third paring, /dada/, was also created to serve as a control condition where there was no acoustic change between S1 and S2. Each stimulus was made by concatenating the two speech tokens using the Sound program in Compumedics Neuroscan Stim2.

Werker and Lalonde [13] created five formant stimuli and constructed a synthesized 16-step continuum by varying the starting frequency of F2 and F3 (second and third formants). The three speech tokens /ba/, /da/, and /Da/ selected in the present study represented equal step intervals across articulation locations and they were equivalent to the 3rd, 8th, and 13th tokens among the 16 steps, respectively. The fundamental frequency was 100 Hz for the first 100 ms then rose to 120 Hz. F1 rose from 250 to 500 Hz over a period of 50 ms while F4 and F5 remained constant at 3500 and 4000 Hz, respectively. The steady-state frequency was 1090 Hz for F2 and 2440 Hz for F3, and the transitions for both F2 and F3 lasted 50 ms. The starting frequency of F2 varied for /ba/, /da/, and /Da/, and they were 1000, 1250, and 1500 Hz, respectively. The starting frequency for F3 was 2384, 2528, and 2627 Hz for /ba/, /da/, and /Da/. Small and Werker [4] reported no significant differences in amplitudes and latencies of the P1, N1, P2, and N2 components elicited to /da/, /ba/, and /Da/ individually in adults except for N1 which had a larger amplitude to /da/ and /ba/ compared with /Da/.

In Small and Werker [4], S1S2 stimuli had a total duration of 564 ms (S1 and S2 were each 282 ms in duration) and were presented with an interstimulus interval (ISI) of 2200 ms. For the current study, the vowel portion for each of the original tokens was lengthened to a maximum 410 ms using Praat 5.3.23 software (vowel durations greater than 410 ms became distorted and sounded unnatural). The total stimulus duration for S1S2 in the present study was 816 ms (Figure 1). The longer S1S2 stimuli were presented with the same ISI as in the previous study; however, the onset-to-onset duration was necessarily increased.

The stimuli were presented at 86 dB peak SPL in the sound field. The stimuli were presented by Stim2 and then delivered to Tucker Davis Technologies PA5 and SM5 modules. The overall gain of the stimulus was reduced by 13 dB before routing it to the HB7 headphone driver which was connected to a loudspeaker placed one meter in front of the infant participant. A Larson Davis System 824 and Larson Davis Model 2559 0.5-inch random-incidence microphone placed at the approximate position of the infant's head were used to calibrate the speech stimulus in dB peak SPL.

### 2.3. Recordings

A four-channel electrode montage was used to record the ERPs in all participants. Individual gold-plated cup electrodes filled with electrode paste were placed at Cz, C3, M1, M2, and FPZ (International 10–20 system) and secured with tape. (Note: only the waveforms recorded at C3 that are presented as the responses at Cz were very similar.) M2 was chosen as the reference and the electrode located on the forehead served as ground. Eye-blink activity was monitored using bipolar electrodes pasted above and below the centre of the left eye. The Compumedics Neuroscan Synamps2 and SCAN 4.3 software were used to record the electroencephalograph (EEG). All interelectrode impedances were measured and kept below 5 kOhms with the SCAN 4.3 impedance routine.

**Figure 2:**
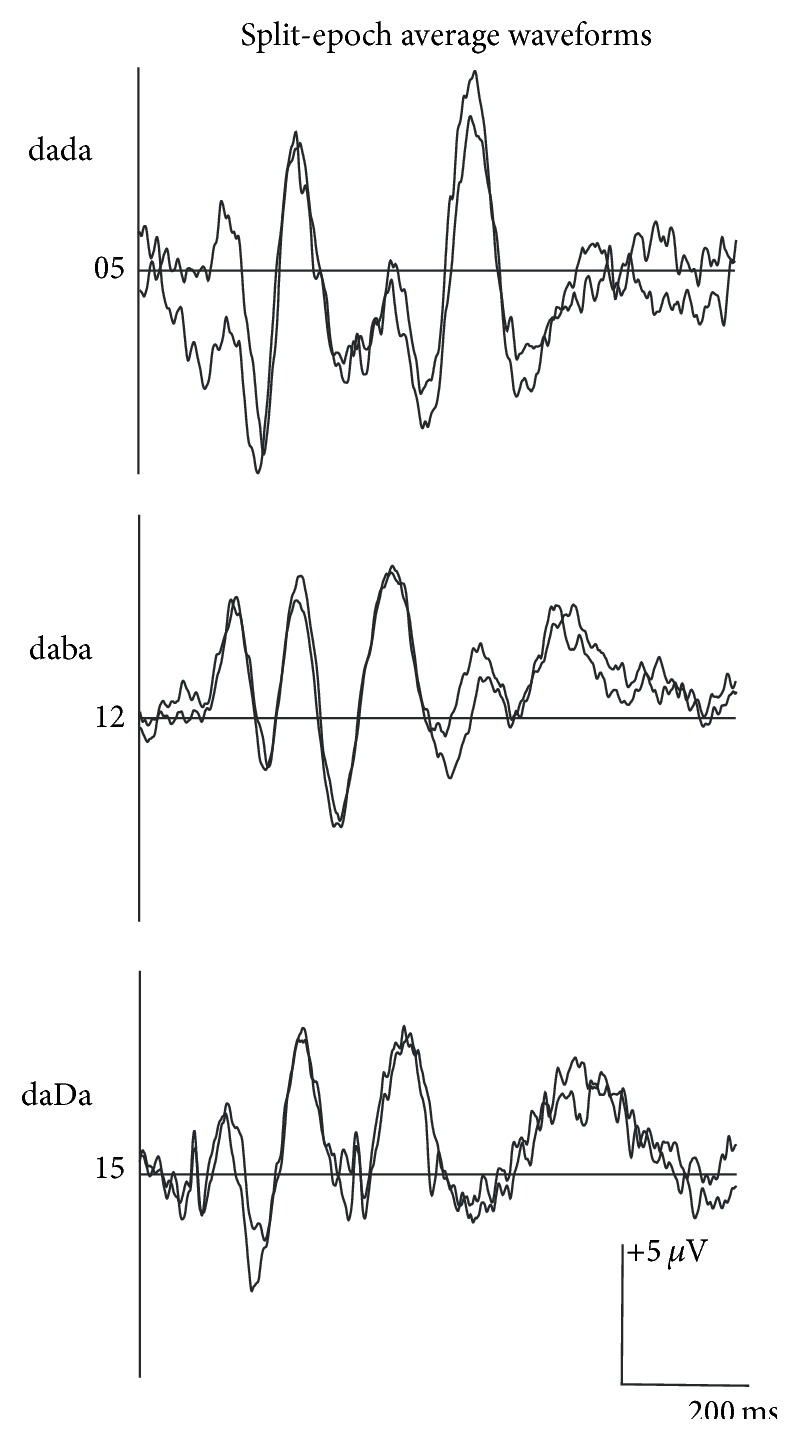
Split-epoch waveforms elicited to /dada/, /daba/, or /daDa/ for an individual infant participant for each stimulus condition.

During data acquisition, the EEG channels were filtered using a 30 Hz low-pass filter and a 1.0 Hz high-pass filter. The continuous EEG was amplified with a gain of 500 and converted using an analog-to-digital rate of 1000 Hz. The recording window consisted of a 100 ms prestimulus period and a 1400 ms poststimulus period. After acquisition, offline analysis used an epoch 900 ms in length (−100 prestimulus to 800 ms poststimulus). Single trials were baseline corrected across the entire sweep duration and an ocular artifact reduction was applied using an average of three epochs, which contained ocular movement greater than 250 ms epoched over −100 to 300 ms. Single trials were rejected automatically for adults when electrophysiological activity exceeded 75 *μ*V in amplitude over a range of −100 to 800 ms; single trials were rejected manually by visual inspection for infants to optimize the number of accepted epochs in the final waveform. A minimum of 130 accepted epochs was required for each stimulus condition to be included in final data analysis based on pilot data for 4-month-old infants; fewer than 130 accepted epochs resulted in poorer morphology and replicability of averaged waveforms. The total number of recorded epochs ranged from 300 to 371 and from 260 to 483 for adults and infants, respectively. The average rejection rate was much lower for adults (5–8%) versus infants (43–48%) and resulted in 244–365 and 135–277 accepted epochs for adults and infants, respectively. A split-epoch method was used to generate two replications (i.e., odd- versus even-numbered epochs) for each condition tested.  Figure 2 shows an example of split-epoch average waveforms for each stimulus condition for individual infants.

### 2.4. Procedure

All tests were carried out in a double-walled sound-attenuated booth in the Pediatric Audiology Lab at the University of British Columbia. Adult participants were seated comfortably in an armed reclining chair and watched a movie with subtitles with no sound throughout testing. They were instructed to ignore the stimuli presented to them and to remain as quiet and still as possible. The infant participant was held by a parent who sat in a comfortable chair facing a loud speaker. An age-appropriate movie was played silently on a flat-screen monitor placed directly behind the loud speaker. An assistant also stayed in the booth to engage the infant's attention in order to minimize the head movement and reduce myogenic noise in the EEG.

Adults were required to complete all three stimulus conditions (i.e., /dada/, /daba/, and /daDa/) to be included in the study, while infants were required to complete at least one condition. The order of stimulus conditions was randomized for each participant. Only five infants completed more than one stimulus condition and none of them completed all three conditions. An experimenter observed the EEG during data acquisition to monitor the infant's state, muscle movement, and electrical artifact. Testing was stopped if the infant started to cry or vocalize continuously during the recording. Hearing screening was conducted in both ears at the end of the test session. The duration of the recording was approximately 1.5 hours for adults and 10 to 40 minutes for infants. After explaining the study to the adult participants and the parents of infant participants, written consent was obtained. An honorarium was given to the adult participants. A small honorarium and a gift were given to the parents and their infants at the end of the session.

### 2.5. Data Analysis

The morphology of ERP waveforms to /dada/, /daba/, and /daDa/ was compared qualitatively and the percentage of components present for each condition was calculated. The baseline-to-peak amplitude and latency of the largest peaks within each of the expected latency windows were also measured. Response latencies were measured for cortical components to S1 and S2 from the onset of S1 and the onset of S2, respectively. For amplitude measures, when the baseline was not at 0 *μ*V for the S2 conditions, the amplitude of P2 was measured from the negative trough preceding P2. Mean latency and amplitude values were calculated for each of the slow cortical components in response to the S1 and S2 portions of the stimulus. Peak-to-peak amplitudes for N1-P2 were also measured for adults and compared for responses elicited to S2 versus S1. Grand mean ERP waveforms to each of the stimulus conditions were also compared in terms of morphology, amplitudes, and latencies.

For the adult group, two-way repeated-measures analyses of variance were carried out to compare (i) baseline-to-peak amplitudes and latencies of the P1, N1, and P2 components elicited to the S1 versus S2 portion of /dada/, /daba/, and /daDa/ and (ii) N1-P2 peak-to-peak amplitudes for S1 and S2 stimuli across stimulus conditions. For the infant group, two-way mixed-model analyses of variance were used to compare baseline-to-peak amplitudes and latencies of the P1 and N1 components evoked to the S1 versus S2 portion of /dada/, /daba/, and /daDa/. Newman-Keuls* post hoc* comparisons were performed for significant main effects. Results for all analyses were considered statistically significant if *p* < 0.05.

## 3. Results

### 3.1. Adults

**Figure 3:**
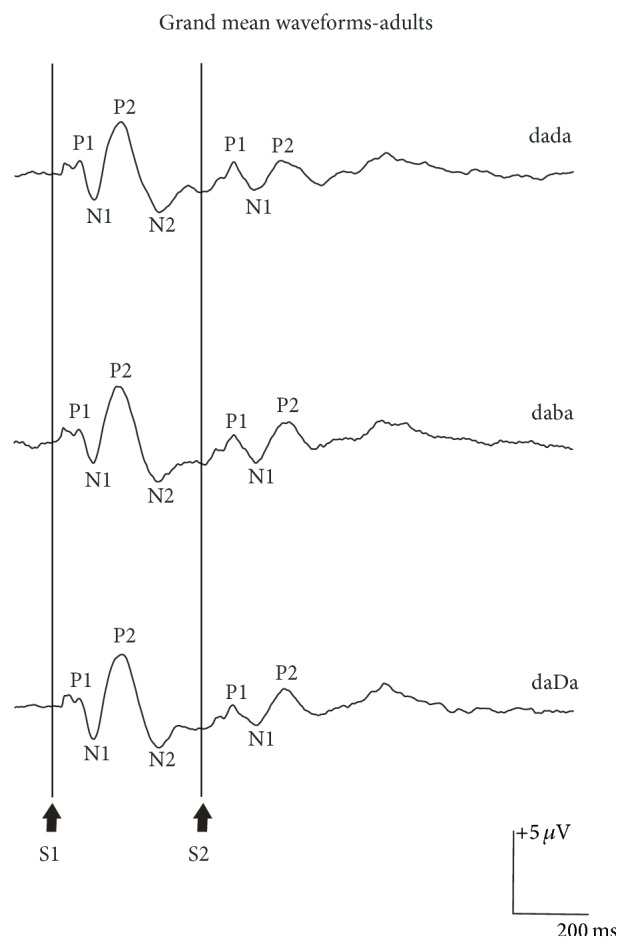
Grand mean waveform elicited to /dada/, /daba/, and /daDa/ at C3 for 10 adult participants. The onset of the S1 and S2 portion of the S1S2 stimuli is shown at the bottom of the graph. The P1, N1, P2, and N2 components of the obligatory cortical response elicited to S1 are indicated on the graph.

As shown in Figure 3, robust P1, N1, and P2 components elicited to the onset of S1 were observed in all three conditions. A similar pattern with smaller amplitudes was also recorded for S2 and presumably in response to the acoustic change. As indicated in Table 1, clear P1 and N1 components were recorded in 90% of the adult participants, while P2 and N2 were present in all adults for S1 stimuli. Mean latencies elicited to S1 stimuli across stimulus conditions were 66, 110, 183, and 293 ms for P1, N1, P2, and N2, respectively, as indicated in Table 2. The mean baseline-to-peak amplitudes for the P1, N1, P2, and N2 components elicited to S1 stimuli across stimulus conditions were 1.92, −2.79, 5.34, and −3.67 *μ*V, respectively.

The overall morphology of the waveforms recorded in response to the acoustic change from S1 to S2 (i.e., the ACC) was similar when compared across stimulus conditions /dada/, /daba/, and /daDa/ (Figure 3). For S2, the P1 component was present in all adult participants, while N1 was recorded in the majority of cases for /dada/, /daba/, and /daDa/; P2 was present in 80% of adults for /daba/ and /daDa/, but only half of the participants showed a clear P2 component for /dada/. N2 was absent in more than half of the adults. The mean latencies for S2 stimuli were on average 89, 168, and 254 ms for P1, N1, and P2, which were 23, 58, and 71 ms longer in comparison to the components evoked to S1. There were no statistically significant amplitude or latency effects for N1 or P2 with the exception that P2 amplitudes were larger and N1 latencies were longer for S2 versus S1 stimuli (Table 3). Comparisons of the peak-to-peak amplitudes for the N1-P2 complex for S1 versus S2 across stimulus conditions revealed that /daba/ was 11-12% larger compared to /dada/ and /daDa/, but this difference did not reach statistical significance [*F*(2,8) = 0.402, *p* = 0.682]. Similar to the pattern for individual components, the mean peak-to-peak amplitude of the N1-P2 elicited to S2 was smaller compared to S1 [*F*(1,4) = 8.251, *p* = 0.045].

### 3.2. Infants

**Figure 4:**
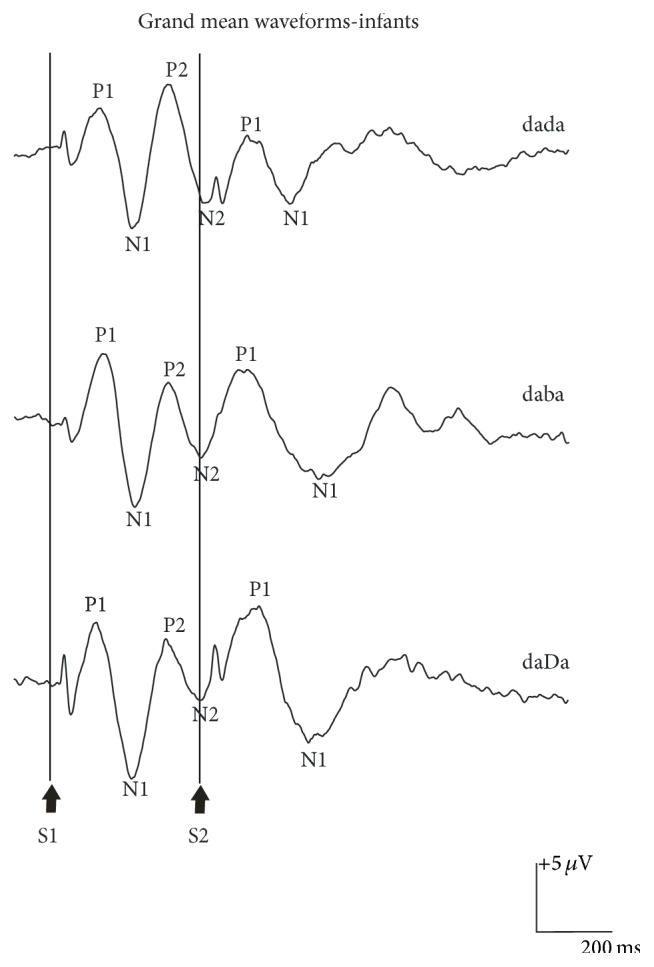
Grand mean waveform elicited to /dada/, /daba/, and /daDa/ at C3 for a total of 24 English-speaking 4-month-old infant participants with normal hearing. The onset of the S1 and S2 portion of the S1S2 stimuli is shown at the bottom of the graph. The P1, N1, P2, and N2 components of the obligatory cortical response elicited to S1 are indicated on the graph.

**Figure 5:**
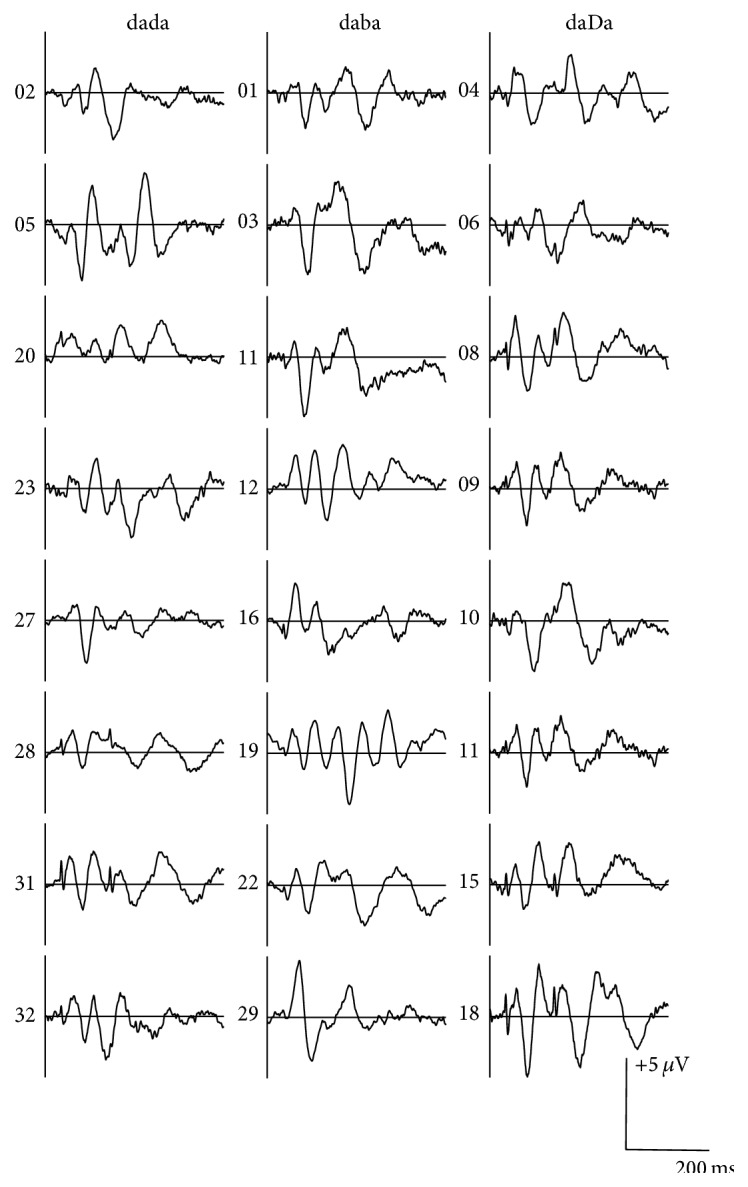
Individual waveform elicited to /dada/, /daba/, and /daDa/ at C3 for the 24 infants described in Figure 4.

Waveform morphology was similar across conditions for the infant participants, as shown in Figure 4. Cortical responses from each of the 24 infants are shown in Figure 5. Similar to adults, cortical responses from most infant participants showed a P1-N1-P2 complex in response to the onset of the S1 stimuli; however, the P1 and N1 components were more prominent and the peak latencies were 26–222 ms later compared to the adult waveforms. A robust P1 was present in all cases and only 12% of the infants failed to show either a clear N1 in response to /dada/ or a clear P2 to /daba/. An N2 component was also found in 63–88% of the infants (Table 1). As shown in Table 4, mean amplitudes for the responses elicited to S1 were larger in comparison to adults and ranged from −9.30 to +7.46 *μ*V; mean latencies across stimulus conditions for P1, N1, P2, and N2 were, on average, 132, 226, 320, and 415 ms, respectively.

The morphology of the grand mean waveform elicited to the acoustic change from S1/da/ to S2/da/, /ba/, or /Da/ resembled the morphology of the P1, N1, and P2 components of S1 responses (Figure 4); however, variability in the morphology and latency of the components was observed when the participants' waveforms were examined individually (Figure 5). All infant participants except one showed a robust P1 component and the mean peak latencies were 136, 134, and 142 ms for /dada/, /daba/, and /daDa/, respectively (Table 4). In contrast, the N1 component elicited to S2 was more variable. A clear N1 component was recorded in the majority of the participants (75–88%), but the grand mean waveform obscured some of the individual differences, resulting in later latencies and broader negative troughs for /daba/ and /daDa/ in comparison to /dada/. The mean peak latencies of N1 elicited to S2 were 259, 285, and 296 ms for /dada/, /daba/, and /daDa/ indicating that S2 responses occurred 34, 55, and 74 ms later compared with S1 responses. For each of the stimulus conditions, the N1 component elicited to S2 either was absent or resembled a broad negative peak in 1-2 infants while the remaining infants had N1 peaks that varied in latency (more variability than what was observed for P1). A second positive peak that resembled P2 was also present in five out of eight infants for /dada/; however, P2 was present for fewer than half of the infants for /daba/ and /daDa/ and the latencies were more variable compared to /dada/. As a result, only /dada/ had a discernible S2 P2 peak in the grand mean waveform (Tables 1 and 4).

**Figure 6:**
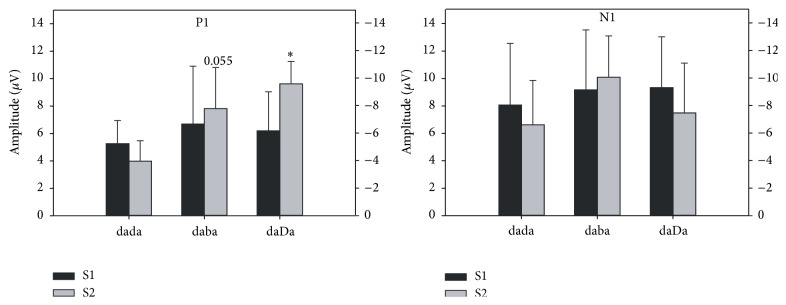
Infant: baseline-to-peak amplitude of the P1 and N1 components elicited to the S1 and S2 portion of the /dada/, /daba/, and /daDa/ stimulus conditions is shown. An asterisk (*∗*) denotes the statistical significance of results (*p* < 0.05); “0.055” denotes a* p* value that approached significance.

The results of a two-way mixed model ANOVA comparing the mean amplitudes of P1 and N1 elicited to S1 and S2 across the three stimulus conditions for infants are summarized in Table 5. There was a significant main effect of stimulus condition for P1 amplitudes, as shown in Figure 6, which was explained by a significantly smaller amplitude for /dada/ compared to /daba/ or /daDa/. The amplitude effect for S1 versus the S2 stimulus was marginally significant and was explained by a larger mean P1 amplitude for the S2 condition. A significant interaction between S1 versus S2 and stimulus condition was also revealed for P1 amplitudes. The P1 amplitude elicited to S1 was larger than P1 elicited to S2 for /dada/, while the opposite pattern was found for /daba/ and /daDa/ (Figure 6).* Post hoc* comparisons using the Newman-Keuls test showed that the amplitudes of S2 P1 were significantly larger for /daDa/ versus /dada/ (*p* = 0.003) and marginally significantly larger for /daba/ versus /dada/ (*p* = 0.055). No statistically significant differences were revealed for P1 latencies.

In contrast to P1, N1 had the largest mean amplitudes elicited to both S1 and S2 across all stimulus conditions (Table 4), indicating its prominence in the morphology of the slow cortical responses recorded in infants. Although the amplitude of S2 N1 was larger for /daba/ than for /dada/ and /daDa/ by visual inspection, a two-way mixed-model ANOVA did not reveal significant effects for N1 amplitudes. There were also no significant latency effects for N1 except that latencies were later for the responses to S2 versus S1.

## 4. Discussion

The results of the present study using a long stimulus duration for /dada/, /daba/, and /daDa/ resulted in similar ACC findings for adults and more robust ACCs in infants compared to Small and Werker [4]. In the present study, the differences in ACC magnitude across stimulus conditions for the adults were not statistically significant, although the N1-P2 grand mean ACC tended to be slightly larger for /daba/, similar to the findings of Small and Werker [4]. For infants, the morphology of the ACC was more complex for all three stimulus conditions. Robust P1 and N1 components were present in the majority of the participants, while fewer infants had a clear P2. For the /dada/ condition, most of the infant participants had the different components of the ACC, which were similar to the P1-N1-P2 complex recorded at the onset of the S1 token. Fewer infants had all three components of the ACC in response to /daba/ and /daDa/, and the morphology of the ACC elicited to these stimuli was more variable compared with ACCs to /dada/; in these cases, a broad negative peak or positive peak occurred at approximately 319 to 605 ms instead of distinct N1 and P2 peaks appearing earlier in the waveform between 179 and 510 ms. The amplitude of P1 elicited to the S2 token of the control condition /dada/ was significantly smaller compared with that of the S2 P1 to the experimental stimulus conditions /daba/ and /daDa/, suggesting that the brain discriminated between the control /da/ and the experimental S2 tokens.

Small and Werker [4] reported that only the infant ACC elicited to /daba/ consisted of P1, N1, and P2 components in their study, while the cortical response to the S2 of /dada/ and /daDa/ were comprised primarily of broad positive and negative peaks. The findings of the present study suggest that, by extending the stimulus length, allowing longer time to accommodate the longer neuronal refractory period for infants, better-defined components of the ACC can be recorded and the overall morphology of the grand mean waveforms are improved. Research has shown that age-related changes in myelination, synaptic refinement, and cortical fiber density underlie the maturation in latency, amplitude, and refractoriness of the cortical component [39, 40]. The formation of myelin along the axon increases the conduction velocity of a signal in transmission, and consequently affects the timing of subsequent signal propagation [41]. Because the latency and synchrony of the neuronal signal are affected by myelination, the evoked potential will have shorter latency, increased amplitude, and more defined waveform morphology with maturation [42]. Incomplete myelination and synaptogenesis will lead to longer neuronal refractory periods and lower cortical excitability in the immature central auditory system [32]. Despite these immaturities, Martin [6] and Martin et al. [7] found that it was more efficient to elicit an infant ACC to the vowel contrast /ui/ for an ISI of 250 ms compared to 500 and 1000 ms. They also found that presentation of a stimulus that continuously alternated was more efficient than an interrupted stimulus. However, they did not investigate separate components of the ACC or the effects of stimulus duration. Our results suggest that long-duration stimuli are needed to elicit robust ACCs with distinct components in infants (and possibly young children), at least for consonant contrasts.

Our findings support that the infant's brain can detect a change in the stimulus from /da/ to /da/, /ba/, and /Da/. Moreover, the larger P1 amplitudes recorded for /daba/ and /daDa/ may suggest that the brain has noticed that the acoustic change from /da/ to /da/ was smaller than the change from /da/ to /ba/ and from /da/ to /Da/. In our hypothesis, we had predicted that the ACC for both /daba/ and /daDa/ would have larger amplitudes and more distinct components compared with the ACC to /dada/ because behavioral studies had shown that English-learning infants under 6 months of age were able to discriminate the native /daba/ and nonnative /daDa/ contrasts [13, 43, 44]. Our findings revealed that the P1 amplitudes elicited to S2 of the experimental conditions /daba/ and /daDa/ were indeed significantly larger than that of the control condition /dada/, which supported our hypothesis. This result is consistent with other research findings, which have shown that speech tokens can evoke distinct neural response patterns; for example, synthesized voiced tokens have been reported to evoke responses that are larger in amplitude when compared with responses evoked by voiceless stimuli [11, 45].

Interestingly, our adult group did not show the same significant differences in the amplitudes of ACC components that we found for the infant group. We had expected a larger difference between the ACCs to /daba/ and the other two stimuli because both /dada/ and /daDa/ should have acted as “control” stimuli for the adults. A contributing factor might have been that the stimulus parameters that were more optimal for infants were too long for adults. The speech stimulus used in the present study consisted of two consonant-vowel structures (CVCV), which was different from the typical CV (e.g., /da/) or VV (e.g., /ui/) stimulus used to elicit cortical responses. Although we only focused on the acoustic change between two CV syllables, the brief transition from consonant to vowel within a CV token may have also evoked cortical responses resulting in overlapping cortical waveforms thus affecting the overall morphology of the ACC [46]. Perhaps the longer duration of the CV syllable was not perceived as one syllable by the adults, so that cortical responses evoked by the brief change from the consonant to the vowel within a CV syllable affected the response to the change from /da/ to the other speech tokens.

There are some limitations to the current study. As mentioned above, the nonnative speech token might have reduced the impact of auditory experience. For example, a MMN study using similar Hindi speech contrasts reported that the magnitude of the MMN can be significantly affected by the order of stimulus presentation (i.e., the magnitude of the MMN is larger when /da/ is the standard stimuli and when /Da/ is the deviant) [47]. Therefore, there may be an order effect, that is, the amplitude of the ACC elicited to /daba/ may be different when compared to /bada/ which we did not assess. Also, we only investigated one set of consonant contrasts so we cannot rule out a stimulus effect; ACCs to a range of different contrasts should be assessed to confirm that this tool is an accurate index of discrimination capacity.

## 5. Conclusion

The most important finding of the present study is that an ACC to a change within a speech stimulus can be successfully recorded in young infants, and, by extending the stimulus length and allowing more time to accommodate the longer neuronal refractory period for infants, better-defined components of the ACC can be elicited. Our ACC results also suggest that distinct neural response patterns may be elicited to acoustic changes that vary in degree. In the present study, ACC components had larger amplitudes in response to a larger acoustic change within a stimulus. To confirm that the ACC is sensitive to a range of subtle acoustic changes in speech, more research is needed. As a technique in development, the ACC may hold promise for providing insight into the infant brain's capacity to discriminate the acoustic features of speech.

## Figures and Tables

**Table 1:** Percentage of responses present for each stimulus condition for infant (*N* = 8) and adult (*N* = 10) participants. The terms “BP” and “BN” denoted “broad positive” and “broad negative” peaks, respectively.

	Token	Component	Stimulus condition		
			/dada/	/daba/	/daDa/
Infant	S1	P1	100	100	100
		N1	88	100	100
		P2	100	88	100
		N2	88	63	75
	S2	P1	100	88	100
		N1	75	75	88
		P2	75	50	38
		N2	25	38	13
		BP	0	13	38
		BN	38	13	25

Adult	S1	P1	90	100	100
		N1	90	90	90
		P2	100	100	100
		N2	100	100	100
	S2	P1	100	100	100
		N1	70	90	80
		P2	50	80	80
		N2	40	50	30

**Table 2:** Adults: mean (1SD) baseline-to-peak amplitude and peak latency measurements for individual components of the waveform elicited to the S1 and S2 portion of the /dada/, /daba/, and /daDa/ stimulus conditions are shown. The latencies were measured from the onset of the S1 stimulus and from the onset of the change in the stimulus at S2. Mean (1SD) N1-P2 amplitudes are also indicated. Mean values that represent measurements from fewer than five responses are denoted with an asterisk (*∗*). The dashed line indicates that no responses were detected. The terms “BP” and “BN” denoted “broad positive” and “broad negative” peaks, respectively.

	Peak	Mean amplitude in *µ*V (1SD)			Mean peak latency in ms (1SD)		
		/dada/	/daba/	/daDa/	/dada/	/daba/	/daDa/
S1	P1	1.86 (1.01)	2.08 (1.54)	1.82 (0.83)	68 (18)	64 (15)	65 (15)
	N1	−2.97 (1.29)	−2.30 (1.30)	−3.24 (2.50)	111 (7)	107 (15)	111 (7)
	P2	4.93 (2.65)	5.70 (2.70)	5.38 (2.58)	183 (14)	181 (19)	184 (18)
	N2	−3.43 (1.08)	−3.85 (1.73)	−3.74 (1.73)	291 (19)	299 (30)	294 (27)
	*N1-P2*	7.49 (3.42)	7.93 (3.20)	8.62 (4.10)			

S2	P1	1.45 (0.72)	1.40 (0.65)	2.56 (0.97)	86 (43)	65 (32)	136 (71)
	N1	−2.21 (0.40)	−2.16 (1.72)	−2.19 (0.53)	155 (63)	73 (20)	211 (103)
	P2	2.05 (1.05)	2.85 (1.29)	1.67 (1.23)	225 (18)	226 (17)	310 (124)
	N2	−0.90^*∗*^ (0.58)	−1.39^*∗*^ (1.07)	−0.71^*∗*^ (0.16)	291^*∗*^ (38)	297 (24)	309^*∗*^ (66)
	*N1-P2*	4.08 (1.63)	4.68 (1.84)	3.44 (0.79)			

**Table 3:** Adults: comparisons of amplitude and latencies for the P1 and N1 components of the slow cortical response to S1 versus S2 elicited by /dada/, /daba/, and /daDa/ using two-way repeated measures analyses of variance.

		Source	df	*F*	*p*
Amplitude	N1	Stimulus	2,8	0.888	0.339
		S1/S2	1,4	2.129	0.218
		Stimulus × S1/S2	2,8	0.633	0.555

Latency	N1	Stimulus	2,8	1.242	0.339
		S1/S2	1,4	12.38	0.025^*∗*^
		Stimulus × S1/S2	2,8	1.242	0.339

Amplitude	P2	Stimulus	2,6	0.260	0.779
		S1/S2	1,3	16.197	0.028^*∗*^
		Stimulus × S1/S2	2,6	1.509	0.295

Latency	P2	Stimulus	2,6	2.013	*0.214*
		S1/S2	1,3	7.137	0.076
		Stimulus × S1/S2	2,6	2.017	0.214

^*∗*^Significant (*p* < 0.05).

**Table 4:** Infants: mean (1SD) baseline-to-peak amplitude and peak latency measurements for individual components of the waveform elicited to the S1 and S2 portion of the /dada/, /daba/, and /daDa/ stimulus conditions are shown. The latencies were measured from the onset of the S1 stimulus and from the onset of the change in the stimulus at S2. Mean (1SD) N1-P2 amplitudes are also indicated. Mean values that represent measurements from fewer than five responses are denoted with an asterisk (*∗*). The dashed line indicates that no responses were detected. The terms “BP” and “BN” denoted “broad positive” and “broad negative” peaks, respectively.

	Peak	Mean amplitude in *µ*V (1SD)			Mean peak latency in ms (1SD)		
		/dada/	/daba/	/daDa/	/dada/	/daba/	/daDa/
S1	P1	5.25 (1.69)	6.69 (4.22)	6.19 (2.85)	133 (27)	139 (20)	124 (15)
	N1	−8.03 (4.48)	−9.13 (4.36)	−9.30 (3.69)	225 (16)	230 (24)	222 (28)
	P2	7.46 (3.46)	5.61 (2.67)	6.56 (4.04)	313 (14)	319 (29)	328 (55)
	N2	−5.81 (4.02)	−7.12 (2.37)	−3.37 (2.88)	430 (25)	432 (57)	384 (44)
	*N1-P2*	14.49 (6.77)	14.04 (3.64)	15.86 (6.21)			

S2	P1	3.99 (1.48)	7.81 (3.00)	9.62 (1.63)	136 (25)	134 (36)	142 (63)
	N1	−6.60 (3.22)	−10.05 (6.99)	−7.46 (3.62)	259 (48)	285 (58)	296 (31)
	P2	7.97 (5.43)	4.26^*∗*^ (2.58)	6.00^*∗*^ (5.08)	448 (69)	391^*∗*^ (97)	421^*∗*^ (34)
	N2	−4.28^*∗*^ (1.14)	−4.09^*∗*^ (3.92)	−3.71^*∗*^	584^*∗*^ (206)	492^*∗*^ (124)	563^*∗*^
	BP	—	4.81^*∗*^	6.35^*∗*^ (1.24)	—	577^*∗*^	518^*∗*^ (32)
	BN	−4.92^*∗*^ (0.94)	−10.09^*∗*^	−5.69^*∗*^ (1.05)	537^*∗*^ (159)	319^*∗*^	587^*∗*^ (26)
	*N1-P2*	14.57 (5.48)	13.69 (8.27)	17.50^*∗*^ (10.54)			

**Table 5:** Infants: comparisons of amplitude and latencies for the N1 and P1 components of the slow cortical response to S1 versus S2 elicited by /dada/, /daba/, and /daDa/ using two-way mixed-model measures analyses of variance.

		Source	df	*F*	*p*
Amplitude	N1	Stimulus	2,15	0.278	0.761
		S1/S2	1,15	0.924	0.352
		Stimulus × S1/S2	2,15	0.867	0.444

Latency	N1	Stimulus	2,15	1.035	0.379
		S1/S2	1,15	28.809	<0.0001^*∗*^
		Stimulus × S1/S2	2,15	2.599	0.107

Amplitude	P1	Stimulus	2,20	4.741	0.021^*∗*^
		S1/S2	1,20	4.309	0.051
		Stimulus × S1/S2	2,20	5.350	0.014^*∗*^

Latency	P1	Stimulus	2,20	0.020	0.980
		S1/S2	1,20	0.382	0.543
		Stimulus × S1/S2	2,20	0.566	0.577

^*∗*^Significant (*p* < 0.05).

## References

[1] Kaukoranta E., Hari R., Lounasmaa O. V. (1987). Responses of the human auditory cortex to vowel onset after fricative consonants. *Experimental Brain Research*.

[2] Martin B. A., Boothroyd A. (1999). Cortical, auditory, event-related potentials in response to periodic and aperiodic stimuli with the same spectral envelope. *Ear and Hearing*.

[3] Ostroff J. M., Martin B. A., Boothroyd A. (1998). Cortical evoked response to acoustic change within a syllable. *Ear and Hearing*.

[4] Small S. A., Werker J. F. (2012). Does the ACC have potential as an index of early speech-discrimination ability? A preliminary study in 4-month-old infants with normal hearing. *Ear and Hearing*.

[5] Martinez A. S., Eisenberg L. S., Boothroyd A. (2013). The acoustic change complex in young children with hearing loss: a preliminary study. *Seminars in Hearing*.

[6] Martin B. A. The effects of stimulus alternation rate on the efficiency of the acoustic change complex in infants and toddlers.

[7] Martin B. A., Goldin L. S., Antony R. M. Efficient stimulus presentation strategies for eliciting the acoustic change complex in infants.

[8] Martin B. A., Boothroyd A. (2000). Cortical, auditory, evoked potentials in response to changes of spectrum and amplitude. *The Journal of the Acoustical Society of America*.

[9] Martin B. A. (2007). Can the acoustic change complex be recorded in an individual with a cochlear implant? Separating neural responses from cochlear implant artifact. *Journal of the American Academy of Audiology*.

[10] Martin B. A., Boothroyd A., Ali D., Leach-Berth T. (2010). Stimulus presentation strategies for eliciting the acoustic change complex: increasing efficiency. *Ear and Hearing*.

[11] Tremblay K. L., Friesen L., Martin B. A., Wright R. (2003). Test-retest reliability of cortical evoked potentials using naturally produced speech sounds. *Ear and Hearing*.

[12] Friesen L. M., Tremblay K. L. (2006). Acoustic change complexes recorded in adult cochlear implant listeners. *Ear and Hearing*.

[13] Werker J. F., Lalonde C. E. (1988). Cross-language speech perception: initial capabilities and developmental change. *Developmental Psychology*.

[14] Eimas P. D., Siqueland E. R., Jusczyk P., Vigorito J. (1971). Speech perception in infants. *Science*.

[15] Little V. M., Thomas D. G., Letterman M. R. (1999). Single-trial analyses of developmental trends in infant auditory event- related potentials. *Developmental Neuropsychology*.

[16] Molfese D. L. (2000). Predicting dyslexia at 8 years of age using neonatal brain responses. *Brain and Language*.

[17] Wunderlich J. L., Cone-Wesson B. K. (2006). Maturation of CAEP in infants and children: a review. *Hearing Research*.

[18] Golding M., Purdy S. C., Sharma M., Dillon H. (2006). The effect of stimulus duration and inter-stimulus interval on cortical responses in infants. *Australian and New Zealand Journal of Audiology*.

[19] Čeponienė R., Cheour M., Näätänen R. (1998). Interstimulus interval and auditory event-related potentials in children: evidence for multiple generators. *Electroencephalography and Clinical Neurophysiology/Evoked Potentials Section*.

[20] Gilley P. M., Sharma A., Dorman M., Martin K. (2005). Developmental changes in refractoriness of the cortical auditory evoked potential. *Clinical Neurophysiology*.

[21] Kurtzberg D., Hilpert P. L., Kreuzer J. A., Vaughan H. G. (1984). Differential maturation of cortical auditory evoked potentials to speech sounds in normal fullterm and very low-birthweight infants. *Developmental Medicine and Child Neurology*.

[22] Orlrich E. S., Barnet A. B., Weiss I. P., Shanks B. L. (1978). Auditory evoked potential development in early childhood: a longitudinal study. *Electroencephalography and Clinical Neurophysiology*.

[23] Rotteveel J. J., Colon E. J., Stegeman D. F., Visco Y. M. (1987). The maturation of the central auditory conduction in preterm infants until three months post term. IV. Composite group averages of the cortical auditory evoked responses (ACRs). *Hearing Research*.

[24] Shucard D. W., Shucard J. L., Thomas D. G. (1987). Auditory event-related potentials in waking infants and adults: a developmental perspective. *Electroencephalography and Clinical Neurophysiology/ Evoked Potentials*.

[25] Wunderlich J. L., Cone-Wesson B. K., Shepherd R. (2006). Maturation of the cortical auditory evoked potential in infants and young children. *Hearing Research*.

[26] Rita R., Rinne T., Näätänen R. (2002). Maturation of cortical sound processing as indexed by event-related potentials. *Clinical Neurophysiology*.

[27] Eggermont J. J., Ponton C. W. (2003). Auditory-evoked potential studies of cortical maturation in normal hearing and implanted children: correlations with changes in structure and speech perception. *Acta Oto-Laryngologica*.

[28] Ponton C. W., Don M., Eggermont J. J., Waring M. D., Masuda A. (1996). Maturation of human cortical auditory function: differences between normal-hearing children and children with cochlear implants. *Ear and Hearing*.

[29] Ponton C. W., Eggermont J. J., Kwong B., Don M. (2000). Maturation of human central auditory system activity: evidence from multi-channel evoked potentials. *Clinical Neurophysiology*.

[30] Ponton C., Eggermont J. J., Khosla D., Kwong B., Don M. (2002). Maturation of human central auditory system activity: separating auditory evoked potentials by dipole source modeling. *Clinical Neurophysiology*.

[31] Sharma A., Kraus N., McGee T. J., Nicol T. G. (1997). Developmental changes in P1 and N1 central auditory responses elicited by consonant-vowel syllables. *Electroencephalography and Clinical Neurophysiology/Evoked Potentials Section*.

[32] Surwillo W. W. (1981). Recovery of the cortical evoked potential from auditory stimulation in children and adults. *Developmental Psychobiology*.

[33] Kushnerenko E., Eponiene R., Balan P., Fellman V., Huotilainen M., Näätänen R. (2002). Maturation of the auditory event-related potentials during the first year of life. *NeuroReport*.

[34] Paetau R., Ahonen A., Salonen O., Sams M. (1995). Auditory evoked magnetic fields to tones and pseudowords in healthy children and adults. *Journal of Clinical Neurophysiology*.

[35] Czigler I., Csibra G., Csontos A. (1992). Age and inter-stimulus interval effects on event-related potentials to frequent and infrequent auditory stimuli. *Biological Psychology*.

[36] Sharma M., Johnson P. K. H., Purdy S. C., Norman F. (2014). Effect of interstimulus interval and age on cortical auditory evoked potentials in 10–22-week-old infants. *NeuroReport*.

[37] Picton T. W., Woods D. L., Proulx G. B. (1978). Human auditory sustained potentials. I. The nature of the response. *Electroencephalography and Clinical Neurophysiology*.

[38] Näätänen R., Picton T. (1987). The N1 wave of the human electric and magnetic response to sound: a review and an analysis of the component structure. *Psychophysiology*.

[39] Huttenlocher P. R., Dabholkar A. S. (1997). Regional differences in synaptogenesis in human cerebral cortex. *The Journal of Comparative Neurology*.

[40] Moore J. K., Guan Y.-L. (2001). Cytoarchitectural and axonal maturation in human auditory cortex. *Journal of the Association for Research in Otolaryngology*.

[41] Salamy A. (1978). Commissural transmission: maturational changes in humans. *Science*.

[42] Musiek F. E., Verkest S. B., Gollegly K. M. (1988). Effects of neuromaturation on auditory-evoked potentials. *Seminars in Hearing*.

[43] Werker J. F., Gilbert J. H., Humphrey K., Tees R. C. (1981). Developmental aspects of cross-language speech perception. *Child Development*.

[44] Werker J. F., Tees R. C. (1984). Cross-language speech perception: evidence for perceptual reorganization during the first year of life. *Infant Behavior and Development*.

[45] Steinschneider M., Volkov I. O., Noh M. D., Garell P. C., Howard M. A. (1999). Temporal encoding of the voice onset time phonetic parameter by field potentials recorded directly from human auditory cortex. *Journal of Neurophysiology*.

[46] Ganapathy M. K., Narne V. K., Kalaiah M. K., Manjula P. (2013). Effect of pre-transition stimulus duration on acoustic change complex. *International Journal of Audiology*.

[47] Tsui V. C. K. (2000). *Mismatch negativity cortical event-related potential measures of cross-linguistic phoneme perception [M.Sc. thesis]*.

